# The Public Life of the Prince and Princess of Wales

**Published:** 1889-04-27

**Authors:** 


### THE PUBLIC LIFE AND WORK

OF

### Their Royal Highnesses

THE

### PRINCE AND PRINCESS OF WALES.

*The monthly part for May will contain Special Portraits of their Royal Highnesses. Intending Subscribers should send their names without delay to the Publisher. Subscribers to weekly numbers can be supplied with portraits at 3d. each.*

### Prince, Princess, and People.*

The general impression about a prince and his duties is that the chief of these is, in Mr. Gilbert's words, "to do nothing in particular and to do it very well." His life is supposed to be all play and no work, and while we all religiously impress on our children the doctrine that all play is a very dreary thing, as nauseating to the soul as a diet of jam and nothing else would be to the body, we are not in our inmost souls convinced of it. We ourselves want more jam on our daily bread-and-scrape, we count him happy who has the means stored up of buying jam in the future, and he who is born to an inheritance of the sweets of this life is the object of our envy. "A man who has nothing to do but enjoy himself!"—we cannot utter the phrase without an accent of longing. And this is what most people imagine a prince to be.

### Politics or Philanthropy.

It is to demonstrate the inaccuracy of this idea of the privileges of Royal birth that Mr. Burdett's book, "Prince, Princess, and People," is written. As a matter of fact, one of the chief prerogatives of royalty is incessant labour, increasing in direct proportion to proximity to the throne. In an absolute monarchy, the work is more directly political; in a constitutional kingdom like ours, it is expended more on objects of social improvement—objects, which, after all, do more towards building up a nation in permanent stability than all the schemes of all the diplomatists. Where there is internal corruption, the strongest army, the most elaborate and far-seeing policy will not do more than postpone ultimate decay; while a nation of contented and honourable individuals, bound to their natural leader by the personal loyalty born of respect, is powerful beyond all proportion to its numerical strength. In our habit of speaking of national life and national prosperity, we are apt to forget that nationality is the outcome of individuality; national strength lies not in the accidents of political supremacy, the fighting of parties, or the victories of one or another faction, but in the accumulation of individual health and individual nobleness. As Mr. Burdett says: "It must not be forgotten, as history will doubtless record, that in days of rapid progress like those through which England has passed during the latter portion of the present century, the co-operation of individuals in their own interests under responsible and capable leaders, has tended more to develop and improve the national life than all the legislation which has been enacted put together."

### A Prince's Work.

It is this individual interest, co-operated into beneficent schemes of one sort or another, that the Prince of Wales has done so much to help; and this history of his public appearances in connection with charitable and educational institutions, as well as those great triumphs of mechanical ingenuity in which our age rivals that of the Pharaohs, forms in brief a history of the social progress of England during the last twenty-six years. There are few, if any, great movements tending directly or indirectly to the national welfare that have not had the Prince's countenance, and often his practical help. The writer admits that the variety of the Prince's charitable interests has been a surprise to him in compiling his book; it will be not less of a surprise to the readers of it. It might be thought that such a work as this must needs be didactic and dull; but the very number and variety of the subjects embraced make monotony impossible, and there are not a

* "Prince, Princess, and People. An account of the Social Progress and Development of Our Times, as Illustrated by the Public Life and Work of their Royal Highnesses the Prince and Princess of Wales, 1863-1889." By Henry C. Burdett. With Portraits and Autographs of their Royal Highnesses, and Illustrations of their Norfolk Home. London: Longmans, Green, and Co.

few anecdotes introduced, which lighten the pages considerably.

### Which is Greater—Chancellor or Prince?

Let us take one relating to the ceremony of the Prince of Wales taking his seat in the House of Lords. "The occasion was of historical interest, and will be for ever memorable from the circumstance that three-quarters of a century had elapsed since a similar ceremony had been performed, and on account of this unique feature, that two Archbishops—those of Canterbury and York—also took their seats on the same night in the House of Lords. Of course, the strictest etiquette and State ceremonial were observed, the attention of the curious being especially attracted by the circumstance, that the Lord Chancellor, who presided, remained seated during the whole time with his head covered. Only at the end, when the Prince came forward and shook hands with him, did that high dignitary condescend to rise and uncover."

### Amusing a Stranger.

Here is another story belonging to a period very little later. The incident occurred in the cathedral at Würms on the occasion of the first meeting of the Prince of Wales with the Princess Alexandra of Denmark—a meeting to which, at the time, none of the Prince's companions attached any special importance. "After the necessary introductions had taken place, the Royal party moved off to examine the frescoes and other beauties of the cathedral; and when they had disappeared in the distance, one of the equerries of the Prince noticed a gentleman who had accompanied the Princess' party standing by himself, and looking lonely and in need of some companionship. He therefore determined to throw aside all insular prejudices and do his best to cheer up his brother equerry under the trying circumstances. He succeeded very well, and a great mutual liking sprang up, which ultimately ripened into friendship, although the English equerry was not a little disconcerted when he discovered—as he did in the course of the evening—that the personage he had done his best to amuse and cheer was no other than the King of Denmark."

This reminds one of the amusing experience of Mark Twain, when he mentally took under his protection a plainly-dressed old lady, who turned out to be the Empress Augusta of Germany.

### The Prince Consort's Legacy.

The first public appearances of the Prince of Wales were made in the character of his father's son rather than in his own. The Prince Consort's children, the inheritors of his cultured tastes, have naturally taken an interest in art and literature, and it has formed part of their reverence for his memory to follow in his footsteps, by supporting those objects with which his sympathy was so well known. Thus we find the Prince of Wales appearing two months after his marriage at the Royal Academy dinner, and a short time afterwards at the anniversary dinner of the Royal Literary Fund. To speak at two such festivals was a severe test for so young a man as the Prince then was. On each occasion his audience was a critical one. The average Briton, content to say with the Northern farmer, "I thought he said what he ought to ha' said, an' I coomed away," was not represented. The listeners were men accustomed to give heed to fitness and fluency of diction, grace and appropriateness of presence and gesture; and even had they desired to forget their observant and analytic habits of thought, the training of a lifetime is not so easily laid by. The acceptance with which the Prince's speeches, short and modest as they were, met from these assemblages of clever and experienced men was in itself homage of a very flattering nature.

### Love for Children.

The presentation of the freedom of the City of London, and the award by the University of Oxford of the degree of D.C.L., are matters of courtesy which may be passed over here with brief notice. They are valuable chiefly as giving opportunity for the display of that affection and loyalty which the whole nation felt for the Heir Apparent and his young wife. More important in many senses is the fact that the first personal appearance of their Royal Highnesses in connection with a charitable object was at the opening of the British Orphan Asylum at Slough, an institution which had just then been removed from Clapham. This is the first instance of that interest in children, especially in suffering and unfortunate children, which has been one of the most pronounced characteristics of their Royal Highnesses' career. Going through the history recorded in this book, we find this interest coming out in various ways. Orphanages, schools, children's hospitals, training-ships, reformatories, apprentices' exhibitions—every enterprise and institution that would help in the well-being of youth has had a goodly share of patronage and support. As a matter of statesmanship, the reasonableness and value of this help is obvious. It is well that all who have fallen on evil days should be comforted and assisted; it is fitting that the aged, who have done their share of life's work, and borne their share of life's burdens, should be cared for in their declining days; but in the hands of youth lies the future of the nation, and according as their powers are trained and their characters disciplined, so will they affect, for good or evil, the destinies of their country.

### Varied Interests.

That the Prince of Wales is not, however, narrow in his interests may be guessed from the fact that in 1863, the year of his marriage, from which the present record dates, he extended his patronage, accompanied in many cases by liberal subscriptions, to eight public charities, eight hospitals and asylums, five agricultural societies, eleven learned and scientific societies, including the Royal Society, the Royal Geographical Society, the Royal Asiatic, the Royal Institute of British Architects, and the Society of Arts, of the last of which he became president. His Royal Highness's first act in this capacity was noteworthy, as showing that bent of his mind which has since been fully evinced by the schemes he has promoted for technical education. It was to promote and carry through to a successful issue a fund to defray the expenses of sending British workmen to the Paris Exhibition, with a view to improving their mechanical and technical knowledge, and to making them better acquainted with the arts and manufactures of the various European countries. Three institutions for sailors were patronised, five schools, seven associations for the promotion of sports and pastimes, three for the relief of permanent distress, in addition to the Highland Rifle Association, and the Army Pensioners' and Church Extension Societies.

### The Cotton Famine.

This period was marked by the outbreak of the American Civil War, which, by paralysing the cotton industry, brought almost incredible misery on the operatives in the great industrial centres of Lancashire. Perhaps, as the extent of long-continued strain is most clearly shown by the reaction when the tension is removed, no more touching proof of the terrible suffering endured during the winter of 1862-3 could be adduced than the fact that when at last some bales of cotton were brought to England a number of women, workers in the long-closed factories, gathered round and sang the Te Deum over them. More than half a million of people applied for relief, and the weekly loss in wages amounted approximately to £170,000. Few countries have had to face so formidable a shock to their home industries, and it speaks volumes for the unity of the British Empire that measures were speedily organised by which the poor law system was strengthened by the passing of a special Act, and subscriptions were raised, not only in this country, but in India, Australia, Canada, and other dependencies of the Crown, which exceeded in amount the sum derived from the rates. Indeed, so universal was the sympathy excited, that Germany, France, Turkey, Egypt, Holland, Spain, Italy, Brazil, and even America, as well as many foreign colonies, opened subscription lists, and remitted large sums to the central fund. It is well to record this action of foreign countries, because England is usually so prosperous as to afford no opportunity for such an expression of neighbourly sympathy, and many of us are apt to forget nowadays this spontaneous action of foreigners. Dissatisfaction has, indeed, not unfrequently been expressed by certain classes of the community when the Lord Mayor of the day has put himself at the head of a reciprocal movement, by opening a fund for the relief of suffering caused by some sudden calamity—such as a famine in China, or elsewhere. The Prince was not in the country when the cotton famine commenced, but he took occasion to show his practical sympathy by forwarding a cheque for 200 guineas to the relief fund.

### The Bishop of London's Fund.

The most important incident of the year 1864 was the establishment of the Bishop of London's Fund, for providing for the religious instruction of the constantly increasing population of London. Church accommodation, which was ample when the compact walled City was surrounded by country villages, was utterly insufficient when these villages became some of the most populous parts of the metropolis ; and money to build and endow new churches, schools, and mission-rooms became an absolute necessity, if a large portion of our population was not to run the risk of growing up in practical heathenism. To the fund promoted for this purpose the Prince of Wales subscribed £1,000 ; and it is pleasant to record that during the last twenty-four years no less than £800,000 has been contributed. That so large a sum is necessary for this purpose, and that a larger could be easily and well spent on it, may be inferred from the fact though the diocese of London has been considerably reduced in area since the institution of the fund, its population still receives an annual increase of 40,000 souls. It is gratifying, however, to note that, thanks to the Bishop of London's Fund, grants have been made in aid of 150 permanent churches, of which 139 are parochial, while 132 have now revenues of their own amounting in the aggregate to £46,000 per annum. Thanks to the assistance given to the fund, 250 clergy are employed, besides many lay agents, scripture-readers, and mission women. But though much is done, more remains to do.

### Visits to Ireland.

In 1865 the Prince of Wales visited Ireland, to open the Dublin International Exhibition. Three times has the Prince gone to that "disthressful country," which in 1865 had not fully discovered its woes ; and only on the the last occasion has there been any attempt to let political bias degenerate into personal discourtesy. The attempt was as futile as it was unworthy. We are sure that the acknowledged representatives of Home Rule—who, however earnestly they may desire local autonomy, or even the most complete legislative independence, have not yet expressed the intention of separating their country from the British Crown—would disavow any complicity in showing a petty rudeness to the Prince and Princess of Wales ; and would agree with us in regarding the Irish-American manifesto—which stated that the Prince of Wales was "an alien and an invader, and if he set foot on Ireland merited death" as not only uncivil, but absurd. Happily, however, Irish loyalty and Irish hospitality are not to be destroyed by dynamitard threats. The Prince of Wales has been thrice to Ireland, and the Prince of Wales still lives.

### Hard Work.

The visit of 1865 was successful, and the Prince made a most favourable impression; but the visit of 1868 was a triumph. On this occasion His Royal Highness was accompanied by the Princess, and though during the ten days they spent on Irish soil they were almost worked to death—for everyone wanted to see them, and every institution sought their patronage—there was no doubt of the enthusiasm of the people. It is true that the Prince had, in the most extreme sense, to be all things to all men; that he had to converse intelligently, if not eloquently, on almost every subject under the sun ; that he had, as the *Times* said, "to examine with respectful interest pictures, books, antiquities, relics, manuscripts, specimens, bones, fossils, prize beasts, and works of Irish art" ; but when it was love that demanded these varied attentions it was well worth doing. When the Royal yacht anchored in Kingstown, a graceful compliment was paid to the Princess. She was presented with a white dove as a token of peace. A similar gift had been made to the Queen when she visited Ireland in 1849.

### A Loyal Maid.

One incident, among many, may serve to show the general feeling. A loyal young Irish lady, mounted on horseback, was bent upon riding close up to the Royal carriage to secure a good view of its occupants. Defying all rules and barriers, she dashed through the ranks of the guards and galloped past the Prince and Princess, exclaiming, "Oh ! thank you all ; I have seen them, and shall go home happy now !" The Prince politely raised his hat to this extremely daring intruder, which is certainly the happiest thing he could have done in such an amusing—yet most touching—adventure.

### The Working Men's Exhibition.

The Dublin Exhibition was not the only one that attracted the Prince of Wales' attention in 1865. Two others which he visited are worthy of notice, not so much because of their size or obvious importance, as because they show that the belief in the value of technical education for the youth of the industrial classes to which we have already alluded, is not, as some people think, one of the Prince's recent "hobbies," but a matter which has engaged his attention from his earliest manhood to the present day. In March he visited an exhibition of their work which had been got up by the workmen in South London, and though the visit was private and unofficial, it established the success of an undertaking which had hitherto failed to attract public attention. In May he opened the International Reformatory Exhibition at the Agricultural Hall, and manifested the greatest interest in the exhibits; which served to prove that children, taken even from the criminal classes, may, by judicious training, be developed into useful members of society.

### The North Staffordshire Infirmary.

In 1866 we come to the Prince's first personal appearance in connection with hospitals, and it is noteworthy that the North Staffordshire Infirmary, at Hartshill, the foundation-stone of which he laid, should be a perfect and successful example of the new and much-condemned "pay system." It is identified to a unique extent with the working classes, who not only contribute annually the whole cost entailed by the treatment of themselves, their wives, and children, but who present quite £500 a year in addition, as a free gift to the infirmary, to be expended on behalf of those patients, not of the artisan class, who would otherwise be unprovided for. In 1867 began the Prince's long and intimate connection with St. Bartholomew's Hospital, of which he became president. In it and its work he has always taken a special interest, although there is not a metropolitan hospital of any magnitude which has not at some time or other received some token of his interest and the Princess's.

### The Bible Society.

The work of these early years is interesting as giving a sort of promise of help which later years were to fulfil. Thus we cannot note the fact that in June, 1866, the Prince of Wales laid the foundation-stone of the new buildings of the British and Foreign Bible Society, without connecting it with his pledge, given to this society before he started on his Indian tour, to do all in his power to promote the Christian religion; and his reception when in Hindustan of a deputation from the Christian colony at Tinnevelly, who presented him with a copy of the Bible translated into Tamil. The growth of the Bible Society has been both swift and steady. In 1803, when Mr. Wilberforce and a few friends resolved to establish the society, there were not more than four million copies of the Scriptures in the world, and these were confined to but few languages. When, however, the Prince laid the foundation-stone in June, 1866, the circulation of the Bible exceeded fifty millions and a quarter, printed in one hundred and seventy-three languages and dialects—a result due largely to the work of the society, which had spent six millions of money at that time in the furtherance of its objects.

### The Lifeboat Institution.

In the same year the Prince identified himself with the Royal National Lifeboat Institution—the envy of foreigners and the pride of the British people. This step, combined with his action towards the Trinity House, and his patronage and support of the Merchant Seamen's Orphan Asylum and the Sailors' Home, gives proof of the importance which the Prince rightly attaches to the Mercantile Marine, which is, in truth, the backbone of England's greatness. It may be well to state that the expenditure of the National Lifeboat Institution is upwards of £70,000 per annum, and that awards have been granted to many brave men and women who have assisted in saving lives from shipwrecked vessels, of the money value of nearly £100,000. The interest thus begun has developed into many forms. It has been extended to embrace our important fishing industry, and has given birth to the society for relieving the widows and orphans of fishermen, and has brought a great deal of valuable information before the public through the Fisheries Exhibitions at Norwich and Kensington.

### Charities of the Three Kingdoms.

The Royal Caledonian Asylum, the Benevolent Society of St. Patrick, and the Society of Ancient Britons, have all enjoyed the patronage of the Prince of Wales, so that every division of the kingdom which contributes its quota to the poor of the metropolis may claim to have won his sympathy. Regarding the Society of Ancient Britons, now better known as the High School for Welsh Girls, where poor children of Welsh parents residing in London are educated, it may be worth while to state that it was founded on St. David's Day, 1715, being the birthday of Caroline, Princess of Wales, and George III. became its patron. Princess Charlotte of Wales, whose loss the whole country most deeply lamented, took the greatest interest in the well-being of this society, and at her death the national feeling found expression in a subscription to its funds sufficient to educate fifty additional children. It had its origin in a desire of the people of Wales to show their loyal attachment to the House of Hanover, and has resulted in much practical good work.

### St. Thomas's Hospital.

The most notable public appearance of the Prince and Princess of Wales during 1869 was their presence when the Queen laid the foundation-stone of St. Thomas's Hospital. This hospital was the first prominent example in this country of such an institution built on the pavilion plan, and, therefore, attracted the attention of architects, sanitarians, and philanthropists generally, who were all anxious to see if this method, apparently so extravagant in the matter of ground space, would show results in the cure of patients sufficiently good to justify its general adoption. There can be no doubt that the experiment has justified the hypothesis on which it was founded—that air and light are great remedial agents—and almost all our new hospitals are being built more or less on the pavilion principle, though this must sometimes be modified by the need for large accommodation in a limited space.

### At Earlswood.

The autumn of this year and the spring of the following were spent in an Eastern tour, but the summer of 1870 found the Prince and Princess visiting the Earlswood Asylum for Idiots, the foundation-stone of which had been laid in 1861 by the Prince Consort. Anyone who has visited an idiot asylum must admit that, of all painful sights, that presented by the inmates of such an institution is probably the most harrowing; and especially is this the case with ladies, whose natural love for children makes them particularly susceptible to the saddening influences of such surroundings. The authorities of Earlswood had, therefore, made arrangements to spare the Princess any shock she might experience by confining the inmates to a portion of the buildings which would not be inspected by the Royal party. When this came to the knowledge of Her Royal Highness she dissented from the proposals with characteristic kindliness and devotion, on the ground that she felt not the least disinclination to look upon any form of physical or mental suffering in the alleviation of which she had taken a hearty interest. This action of the Princess has been of very real service to the managers of Earlswood and similar institutions, because they had previously found the greatest reluctance amongst ladies to visit patients, a reluctance which had resulted in an enforced isolation, good neither for the inmates nor the staff.

### The Example of George Peabody.

The bent of the Prince's mind was shown in two ceremonies which he performed in 1870: the unveiling of the statue erected to the memory of George Peabody, the illustrious American who devoted so much time and money to improving the housing of the London poor; and the opening of the Workmen's Industrial Exhibition at Islington. In the same year the Prince formally opened the Thames Embankment, which substituted a splendid boulevard for a number of narrow and insanitary streets. In connection with hospitals must be mentioned the Prince's visit to the Infirmary at Portland Prison—is it not something for a convict to remember that once, and in kindness, not as a representative of authority, the heir to the throne visited him? This year also the Prince laid the foundation-stone of the new Royal Infirmary at Edinburgh, one of the largest and most perfectly appointed hospitals in the kingdom, and the clinical school of a university as famous throughout the world for its medical teaching now as Salerno was in the middle ages. Edinburgh Infirmary contains over 600 beds; occupies a site that would rouse the envy of many Londoners, overlooking an extensive park; and, like St. Thomas's, is an admirable example of the pavilion style of architecture.

### The Value of Festival Dinners.

The unforgotten illness of 1871 put a period to all work, either of pomp or charity, for many months, but with recovery came renewed and increased energy in works of social improvement. The opening of the Bethnal Green Museum, the laying of the foundation-stone of the Children's Hospital in Great Ormond-street—so often visited since by the Princess and her daughters—and a review of boys from several training-ships and from the pauper schools of the metropolis, were the most notable acts of 1872. In 1873 the Prince presided at the annual dinner of the Railway Benevolent Institution, a courtesy which formed a graceful requital of the care and vigilance shown by the railway companies of Great Britain during the frequent journeys of members of the Royal family. The Prince's presidency at such festival dinners is, however, valuable as something more than a matter of courtesy. At the various banquets attended by him in 1871, no less than £25,000 was collected for the various charities they represented; that held in aid of the Artists' Benevolent Fund realising no less than £12,300. In this connection we may mention the trouble shown by the Prince in getting up the Landseer Exhibition in 1873. Landseer was always a favourite with our Royal family and their relations, and among those who lent pictures for the exhibition were not only the Queen, but the King of the Belgians and the Duke of Coburg. Although few painters have won such a general and flattering popularity with all classes as Sir Edwin Landseer, his success never undermined his naturally modest and retiring character. It is said that when, in 1866, he was elected President of the Royal Academy he could not be persuaded to hold the appointment more than a week.

### The Artisans' Dwellings Act.

The year 1875 was marked by a Parliamentary recognition of the growing needs of our large cities—the passing of the Artisans' Dwellings Act. This question of the housing of the poor is one that has always engaged the attention of the Prince of Wales. Those who love him least are forced to admit that he has set a good example to many landlords by the attention he has shown to the comfort and welfare of the workers on his estate at Sandringham; and he has devoted genuine thought and labour to the question of improving the homes of the working classes in London and other large towns. He visited in person some of the worst parts of London and Dublin, and it was as no mere ornamental figurehead that he presided at the Commission which dealt with this important matter. The housing of the poor is a question full of difficulties. Reformers have to fight, not merely against vested interests and the large indifference of the public, but against all the prejudices and traditions of the working-man himself. Mr. Burdett gives an example of this prejudice in the story of a respectable labourer who, when congratulated upon the smart appearance his room presented, owing to its having been recently whitewashed and repapered, remarked: "Aye, sir; but it ain't so comfortable like, because there is no place one can lean against without making a mess and leaving a mark!"

### The Landlord's Plea.

While much of the opprobrium that has been cast on the owners of the dwellings of the poor is deserved, it must not, in fairness, be forgotten that there is another side to the question. Houses, large or small, for rich or poor, are not built for charity. The builder means to have interest for the money he expends, that interest taking the form of rental. If he builds a large house in a good locality, he gets this rent in one sum from one tenant, who will probably send a cheque for the amount, unasked, every quarter. If on the other hand, he builds a block of tenements in a poor locality, he receives his rent from twenty tenants, whose payments, supposed to weekly, are most irregular and uncertain. He has to pay a collector to go to his tenants and demand these sums, and he has always, in his calculations, to allow for some of them never being paid. More from force of circumstance, perhaps, than from native dishonesty, the poor are more in the habit of leaving their rent unpaid than the rich, and more given to those retiring movements known as "moonlight flittings"; and the landlord, in fixing his rents, has to make allowance for these accidents, as well as for the much greater damage done to his property by artisan tenants. These considerations often cause the rent of poor houses to be apparently, though not in reality, disproportionately high; though, when all is said, the statement, so frequently repeated, that the owners of tenements in the poorer parts of the town make more than the possessors of houses in fashionable localities is both absurd and untrue.

### The Training of Tenants.

While, however, we express this opinion, which is founded entirely on personal observation, we feel most strongly that much must be done before the dwellings of the poor can be regarded as meeting the requirements of health and morality; but it is necessary not merely to build suitable houses, but to train up a population that will appreciate them. We are reminded, in this connection, of the sad experience of a Scottish coalowner, who, thinking that his miners were not properly accommodated in the two-roomed houses provided for them, built a row of cottages of four apartments. What was the result? The more economical goodwives immediately took in lodgers; the more extravagant turned three rooms into unused "best parlours," gorgeous with bead baskets, wax flowers, and china lapdogs; while in both cases the whole family for whom the increased accommodation was designed lived in the kitchen as heretofore.

### Preparing for India.

Preparations for the Prince's tour in India occupied a great part in 1875. At the time the proposed visit caused endless discussion, and the wildest prognostications were made as to the motive with which it was undertaken and the results that would accrue. Looking back on that time from the vantage ground of a day when it no longer seems remarkable that a prince of the Royal blood should go to India, and live and work there like any other Briton, we can see that the Prince of Wales's journey was an unmixed good. Personal intercourse is, save in cases like that of Anne Page and Master Slender, the surest basis of friendship; and the acquaintance then made by the Prince of Wales with the native chiefs, developing in many instances into mutual regard, the readiness with which he conformed to native customs, and the tact with which he avoided demanding any homage that would be humiliating to these proud and sensitive Eastern princes, has had the most beneficial results. To His Royal Highness's behaviour may be accredited, at least in part, that deeper sympathy on the part of the tributary monarchs, that consciousness that they form an integral part of a great Empire, which has led them since 1875 to give such loyal aid in frontier difficulties, and to offer their money and their men for the quelling of disturbances elsewhere. It is to be wished, indeed, that other Europeans would imitate the courtesy and consideration for the feelings of others which was shown by the Prince of Wales. In the justice and forbearance of the conquering race, in the respect they show to a civilisation older than their own, and to ideas that are rendered sacred by a thousand associations they cannot understand, are to be found the only safeguards against the horrors of another mutiny.

### The Indian Famine.

As an indirect effect of the Prince of Wales's visit to India, we may hazard the surmise that the renewed interest in all that belonged to our Eastern Empire helped in the goodwill with which subscriptions were sent for the relief of the famine which devastated India not long after his return. Twenty-six millions of human beings were affected by this famine, which taxed to the utmost the resources of the Indian Government and charitable organisations. The fund collected at the Mansion House for the relief of this famine amounted to half a million sterling.

### Interest in Manufactures.

On his return the Prince was soon drawn once more into active public life. His energy was largely instrumental in gaining for Norwich a much-needed new hospital. His visit to Glasgow, in order to lay the foundation-stone of the new post-office, associated him with that splendid scheme of cheap intercommunication between all parts of the globe which Great Britain originated, though Mr. Henniker-Heaton has lately shown us that we have been less vigorous in developing it than other nations. In July, 1878, he, with the Princess, visited Nottingham, to open the Midland Counties Art Museum, now housed within the historic walls of Nottingham Castle. There is scarcely a single centre of industry that the Prince of Wales has not visited, often with the direct intention of inspecting its manufactures. In the first year of his married life he opened the Town Hall at Halifax; he has since visited Sheffield, Leeds, Birmingham—where the Princess helped in the electro-plating of a vase—Leicester, Manchester, Worcester, Luton, the home of the straw-plaiting manufacture, and High Wycombe, famous for its chairs.

### The Death of the Princess Alice.

The end of 1878 was marked by the death of the Princess Alice, Grand Duchess of Hesse Darmstadt, a grief in which the whole country was at one with the Royal Family. The tragic circumstances of her death are too well known to require narration, and her many acts of kindness, her untiring industry, her catholicity of spirit, and her quick intelligence are better known now than in her lifetime. A characteristic story of her life in Darmstadt is worth quotation: "The Princess once intimated to a lady of her acquaintance that she would come to tea on a certain afternoon which was named. The hostess attached the greatest importance to the visit, went to no little trouble and expense in decorations for her rooms and entrance-hall, had scarlet cloth put down in front of her house and up the staircase, and having completed all the arrangements to her satisfaction, she prepared to receive the Princess with due *empressement.* She even sent one of the servants to the top of the house to keep a sharp look-out, so that due notice might be given to everybody of the approach of the Princess. Satisfied that everything had been thought of and adjusted, the hostess repaired to the drawing-room and waited. Suddenly the door opened, and a lady entered, robed in a macintosh, with goloshes and an umbrella. As she entered, she remarked: 'Here I am. It is terrible weather, and I have done my best in coming up-stairs not to make a mess on the beautiful red cloth which I saw in the hall and on the steps.'" It was, of course, the Princess Alice.

### Cabs *versus* Umbrellas.

One of the most interesting of the many benevolent associations with which the Prince is connected is the Cabdrivers' Benevolent Association, of whose very existence many people are ignorant. Cabby is generally supposed to be a miracle of improvidence as well as extortion, and few people are willing to give him his due in the matter of honesty. It must have been a surprise to many of those present at the festival dinner in 1878, at which the Prince presided, to learn from his lips that during that year between sixteen and seventeen thousand articles had been left in cabs, amounting in value to about £20,000, all of which had been promptly returned. The only article a cabman is said never to return is an umbrella. Perhaps, however, this is due less to dishonesty than to business shrewdness. It is said that John Bright once defined adulteration to be a form of competition; and the umbrella undeniably competes against the cab. It is not the provident citizen who never takes his or her walks abroad without being armed with the national weapon who calls a hansom when a shower comes on; and the umbrella may therefore be looked upon as the cabman's natural enemy. An incident which the Prince narrated *àpropos* of the honesty of cabmen is worth quotation. A gentleman who had driven to a shop in a cab had an altercation with the shopman, and in consequence of it came out and walked away, forgetting both his cab and a case of jewellery worth £2,300. A few moments after the shopman came out, and flung the case into the vehicle; and the cabman at once drove to Scotland-yard, and delivered the treasure to the superintendent.

### Hospitals for Consumption.

This year the Prince and Princess showed their interest in the sick by laying the foundation-stone of the Hospital for Consumption at Brompton, which was to double the accommodation then existing for patients suffering from this national scourge. There can be no question that of all hospitals none, as a class, provide better accommodation than those for consumption, whilst it must be borne in mind that the amount of care and treatment which these cases require necessitates a very considerable expenditure. Previous to the enlargement of the Brompton Hospital for Consumption to which allusion is here made, it was difficult and at times impossible to procure a bed in a hospital for consumption. Indeed, it may be said with truth that the people's necessities demand a greater number of consumption hospitals of the first class than already exists, and the Prince of Wales was quick to recognise and emphasise the fact by attending the laying of the foundation-stone of the Brompton Hospital. His Royal Highness further enforced his conviction on this point by presiding at a festival dinner in aid of the Royal Hospital for Diseases of the Chest some four years later, when subscriptions amounting to nearly £5,000 were received, the Prince himself subscribing 100 guineas.

### Seniores Priores.

Towards the end of the season their Royal Highnesses the Prince and Princess of Wales once more manifested their interest in the welfare and training of children by going to Greenwich, accompanied by their two sons and the Duke of Cambridge, to distribute the prizes to the boys at the Royal Hospital Schools. An incident connected with the proceedings on that occasion is worthy of remembrance. When the Royal visitors were assembled on the platform in the hall where the distribution of prizes was to take place, it was discovered that there was one chair too few for the Royal party, who were, of course, alone to be seated. The Duke of Cambridge insisted, therefore, on standing, although the two young Princes remonstrated with him. Prince George, however, after occupying his chair for a minute, could endure it no longer. Being an Englishman as well as a prince, he felt that seniority has claims stronger than a relatively greater proximity to the throne. He might in strict etiquette take precedence of his cousin and namesake; but he could not forget that he was a boy, while the Duke of Cambridge was a man of mature age. So he ran off to the chair which Prince Albert Victor occupied, where he made him make room so that the two boys could occupy the same seat. This done, he made a pleasant grimace at the Duke, who sat down in the vacant chair amidst general cheering.

### Hunstanton Convalescent Home.

An instance of the stimulus to good works which Royal personages have it in their power to give by the mere force of example was afforded at the opening of the Hunstanton Convalescent Home, situated only a few miles from Sandringham, which was founded in commemoration of the Prince's recovery. On this occasion the Princess rang the great bell, now used to summon the patients to meals, as a signal that the new buildings had been successfully inaugurated and were ready for occupation. She was, of course, greatly interested in the success of this establishment, and when there was some difficulty about providing the necessary furniture and fittings she at once came forward and gave the cost of one of the forty beds. Immediately thirty-nine other persons came forward to give one bed each, and so the new convalescent home was well and thoroughly furnished without delay or difficulty.

### The Princess Helena College.

About this time the Prince identified himself with the institution formerly known as the Adult Orphan Asylum, and now—having been developed both in size and usefulness through the energy of the Princess Christian—as the Princess Helena College. It was founded in 1818 by a relative of Lord Sydney, who was aided by the Princess Augusta of Gloucester, for the purpose of educating the daughters of clergymen and officers of the army and navy. It is needless to say that the education deemed sufficient for young ladies in 1818 is triviality itself compared with that of the girl of our days. Conducted on its original lines, the institution must have decayed; but developed into a modern school, its funds supplemented by a number of paying pupils, and its situation transferred from Regent's Park to Ealing, it is both useful and successful.

### Women's Hospitals.

Among other institutions which modern care for women and their needs has brought into being is the hospital for women's diseases. Whether the need for them is greater than it was in past generations, or is only more generally recognised; whether or not there is anything in the education and general upbringing of the girls of the present day that renders their womanhood more liable to a number of dangerous and painful diseases, it is difficult to say. And to whatever side opinion leans there are very few who do not feel that these institutions are doing needful and valuable work. The names of the Prince and Princess of Wales are connected with the chief hospitals for women in London. The first with which they actively identified themselves was the Hospital for Women in Soho-square, one of the oldest and best of the class. Later, when new arrangements gave the Chelsea Hospital for Women a fairer chance of existence than it had hitherto had, the Princess laid the foundation-stone of the new building, and patronised an "Old English Fayre" held in aid of its funds in the Albert Hall, which aroused great interest at a time when costume bazaars were rather less common than at present. The year after (1881) the Prince presided at a dinner in aid of the Hospital for Women and Children in Waterloo-bridge-road, the oldest hospital of the kind in the kingdom. It was founded in 1816 by the Duke of Kent, Her Majesty's father, and had therefore a special claim on the Prince's interest.

"The Lungs of Crowded Cities."

It must have surprised everyone who has examined a well-preserved castle or mansion of bygone days to find that the windows were not only small, but fixed in such a way that they evidently were meant never to be opened. How did people breathe in those days? how did they ventilate their apartments? The young science of sanitation has advanced with such rapid strides that it is difficult to realise what a very modern thing an appreciation of pure air and clean water is. It is true that William Bullein did point out in Queen Elizabeth's time that it was not quite wholesome to have dead dogs lying in too close proximity to a dwelling-house, and that some country mansions in the time of William and Mary were provided with bathrooms; but the people at large were only too content to do without any but the most rudimentary sanitary appliances. To this ignorance and indifference is due the fact that so many of our great towns were close-packed masses of houses, unprovided with any of those public parks which have been called "the lungs of crowded cities."

Royalty and Fresh Air.

The Prince of Wales—and, indeed, the whole Royal Family—have always been earnest in endeavouring to procure for the people of our cities as many open spaces as possible. Perhaps this is the result of the healthy open-air life of their youth, for "the heated atmosphere of courts" is a phrase that does not apply to Great Britain. They have rarely, if ever, refused to be present at the opening of any sort of park, be it ancient burial-ground, historic wold, or recreation ground laboriously manufactured by pulling down old houses and importing sods, so long as it was destined for the use and healthy enjoyment of the people. The Prince of Wales has opened Jesmond Dene, given to the people of Newcastle by Sir William Armstrong; the park presented to Sheffield by Mr. Mark Firth; the Abbey Park at Leicester; and the recreation ground at Whitechapel. An example thus set is sure to be followed, and there seems every reason to hope that the encroaching on open spaces, which so long went on unchecked, will be permitted no longer, and that as towns increase their growth will be so arranged that none of their inhabitants will be hopelessly remote from grass and trees and flowers.

The Decline of the Apprentice.

With the changes in national custom which have taken place since the century began, there has come loss as well as gain. The old custom of apprenticing a boy to a master in the trade he was to follow was doubtless capable of abuse. Sometimes, even in these days, an employer gets out of an articled pupil—who is almost the same as an apprentice—more than he ought. Mr. Pecksniff added a pump to Martin Chuzzlewit's plan of a school, and claimed it as his own. Still, as all masters were not Pecksniffs, the young Chuzzlewits got, as a rule, a thorough and practical insight into their calling before they began to follow it at their own risk. The fashion of teaching a boy his trade fell out of fashion with the increase and development of popular education. It was held to be enough to train his intellectual faculties, and leave him to apply them as he would in his life's work. This is a very good training for men of genius, who are safe to use their faculties with insight and energy. But the average man, the average boy, is not a genius; you may cram him with facts and general principles to the point of meningitis, and he will never apply them at all. For him a thorough knowledge of any one branch of human industry is more to be desired than a top-dressing of "culture" which he does not know how to use.

Technical Training.

The recognition of this fact has come to us gradually, painfully, as we found that other nations were equalling, rivalling, surpassing us in industries of which we once possessed the monopoly; and the remedy has been found, we hope, in the establishment all over the country of technical schools. Most people have very vague ideas as to what a technical school is. Many think that it confines its operations only to the decorative arts; that in it only wood-carving, fretwork, art needlework, and the like are taught. These things are indeed studied, with admirable results in the improvement of national taste; but a technical school has a far wider range. It embraces every form of handicraft, and attempts to give thorough teaching in all; as witness the great technical school at the Polytechnic, where instruction is given in such unromanti but necessary trades as toolmaking, carriage-building, watchmaking, and a score of others. And these technical schools give to those who attend them a "culture" as sound as can be gained from the study of biology or mathematics; for they teach that to be thorough, painstaking, and conscientious, is essential to success. This is good moral training, and not such bad mental training either.

Arts and Crafts.

In the encouragement of this new development of national life the Prince of Wales has helped. Perhaps the method pursued in his own education, which included carpentering as well as the classics, has taught him that habits of observation and accuracy, as well as the natural growth of manual dexterity, are to be gained from this practical teaching. Certainly it is clear—"Prince, Princess, and People" brings it out very clearly—that all his life he has taken a very special interest in the development of handicraft. He encouraged the earliest efforts of working-men to bring thought and originality, as well as skill, to bear upon their labours; and he has consistently kept up this encouragement to the present time, when the Apprentices' Exhibition at the People's Palace, and the more recent Arts and Craft's Exhibition at the New Gallery, must have proved to him and all—peers, authors, and socialists—who have laboured to the same end, how well directed and successful their efforts have been.

The International Medical Congress.

It would be unpardonable in this journal to omit a brief allusion to the Prince's attendance at the meeting of the International Medical Congress, which was held at St. James' Hall in August, 1881, and to record the appropriateness of the speech he made on that occasion. On this occasion substantial proof of our insular prejudice was offered by the circumstance that no lady medical practitioner was admitted to the meeting, although women doctors had been encouraged to join the Congress in all foreign countries where it had previously taken place. But it is hardly likely that such a piece of discourtesy will again be set down to the discredit of British doctors. The interest felt by the Prince and Princess in the results secured by the art of medicine was shown at this time, by their visit to the Marylebone Infirmary, their interest in the London Fever Hospital, their visit to the Ascot Cottage Hospital, and by the Prince visiting and subsequently becoming patron of the British Home for Incurables. The children came in for a large share of patronage this year, the Prince presiding at a festival dinner at the Victoria Hospital for Sick Children in March; and both being present at the opening of the Brighton Children's Hospital in July, at the laying of the foundation-stone of the Great Ormond-street Hospital in the same month, and at the opening of the St. Leonard's Convalescent Home for Children, of which they became patrons.

### New Docks.

About this time an epidemic of dock-opening befell the Prince of Wales. There were the Alexandra Docks at Newport, the Albert Edward Dock at Newcastle, or, to speak more accurately, Coble Dene-on-Tyne, and a new dock at Swansea. Not long before he had opened a dock at Grimsby and a new harbour at Liverpool. Bad trade or no, the shipping interest of Great Britain seems to go on growing.

### The Abolition of Slavery.

The great philanthropic festival of 1884 was the jubilee of the abolition of slavery in the British colonies. There are some people on both sides of the Atlantic who still protest that slavery was a blessing and not a curse, but they belong to a generation which cannot recall the horrors of the "middle passage" at sea. But they may at least hearken to the testimony of those who have witnessed the characteristics of the slave trade where it still exists, in Central Africa. Livingstone left it on record as his belief that half a million human lives are annually sacrificed by the African slave trade. This horrible traffic runs in three tracks, marked by skeletons, from the centre of Africa towards Madagascar, towards Zanzibar, and towards the Red Sea. Of those who are carried away by force, some are so worn by fatigue that they die; others, falling by the way, are slaughtered with the sword, so that of this great multitude only a third ever reach the end of their journey and arrive at their horrible destination. These same good folks protest that the slaves liked slavery. That rash statement may be disposed of by Mr. Buxton's graphic account of the manner in which the slaves received the proclamation of their freedom: "Throughout the colonies the churches and chapels had been thrown open, and the slaves had crowded into them on the evening of the 31st July, 1834. As the hour of midnight approached they fell upon their knees and awaited the solemn moment, all hushed, silent, and prepared. When twelve o'clock sounded from the chapel bells they sprang upon their feet, and through every island rang great sounds of thanksgiving to the Father of all, for the chains were broken and the slaves were free!" Slavery, however slowly it may die, is a doomed institution; and it is not for the English nation, at least, to dream that the untiring labours of Livingstone, the sacrifice of Gordon, or the heroic efforts of Stanley, have been made in vain.

### The Hospital for Nervous Diseases.

University College Hospital, the Jaffray Hospital for Chronic Diseases at Birmingham, and the all but unique National Hospital for the Paralysed and Epileptic in Queen's-square, came in for the Prince's attention in 1885. The last institution, the scene of many of those miracles of cranial surgery and careful and elaborate treatment of nervous diseases, which have made many diseases curable that were once looked upon as hopeless, had attracted the notice of the Duke of Albany. A wing had been added to the hospital in memory of him, and this fact doubtless had its share in winning the interest of the Prince of Wales. A similar feeling of affection for his brother, then but lately dead, may have prompted His Royal Highness's action in opening Bream's-buildings, in connection with the Birkbeck Institute, an enterprise by which the Duke had been greatly attracted.

### The Kensington Exhibitions.

The great exhibitions that were held between 1883 and 1886 cannot be passed over without mention, though they are of such recent date that no special account of them is needed. Some people insist that they did not afford much information about fisheries, health appliances, inventions, and Colonial and Indian products, but were simply the "Excuseries" Corney Grain spoke of; and merely afforded a pleasant rendezvous for the people of London. If it were but so it were something; but the means of gaining information certainly were provided, and if the average Londoner did not profit by them, the average Londoner—who is a rather languid and blasé animal—has only himself to blame. His country cousins certainly gained something by their visits to the exhibitions, and will sing "God bless the Prince of Wales" with all their hearts when they think of the energy he showed in getting them up. And those of us who have a spark of national pride in our hearts must, in 1886, have felt them thrill in looking down the great hall hung with banners, and rich with the gorgeous colouring of Eastern wares; and in wandering from one section to another, seeing the varied products which, sent from every quarter of the globe, were tokens of Britain's greatness, and part of Britain's wealth.

### Gordon.

The Colonial and Indian Exhibition must be regarded as the chief incident of 1886, but it should not make us forget such works as the inauguration of the Yorkshire College at Leeds, and Sion College on the Thames Embankment, and the foundation of the Gordon Boys' Home. There is no need as yet to speak of Gordon's virtues. A modern Bayard, *sans peur et sans reproche*, he was also one of the pure in heart to whom it is given to see God. The ever-present sense that God was with him was his strongest inspiration, and was the creed with which he tried to imbue the waifs and strays he taught at Gravesend—the creed which others, for his sake, and in memory of him, are trying to teach a new generation of boys, rescued from surroundings that would lead them straight to vice and crime.

### The Torbay Fishermen.

It will be remembered that in the spring of 1886 the health of the Princess of Wales gave way to some extent, and that she spent some time at Torquay to restore it. A pretty incident that occurred at that time is worth recording. The fishermen of the Devonshire coast were very anxious to give expression to their feelings by welcoming the Princess. Her Royal Highness's residence at the time was situated on the right of the Bay, and on February 27 she was surprised to see a fleet of 50 fishing smacks sail over Torbay in a little squadron, and then, directly they came abreast of the Royal residence, all simultaneously dip their flags in her honour. Having done this, the little vessels put about and departed to pursue their daily work, the men being perfectly satisfied that their beloved Princess would now realise how devoted they all were to herself, and how full were their hearts of sympathy for her illness.

The Unemployed.

The close of the year was darkened by great distress among the poor of London. A fund for the relief of the unemployed was organised at the Mansion House, and a large sum was collected; but it is now generally admitted that the lavish alms then distributed did harm as well as good. The subject of the unemployed poor, and the best methods of helping them, have been so often discussed in our columns that it is needless to do more at present than to repeat our conviction that the best results will be obtained by careful organisations distributing limited and prudent charity, within small areas where they have personal acquaintance with the people. Large funds intended to supply the needs of large districts are inevitably liable to abuse, because those who distribute the charity have no real knowledge of those who demand it.

The People's Palace.

The year 1887 is memorable above all things as being the year of Her Majesty's Jubilee; but it was notable as being marked by the realisation of a dream. It was, we think, in 1883 that Mr. Walter Besant began to dream, in the pages of "Belgravia," of a place where the tired and dreary lads and lasses of the East End could go for wholesome amusement and intelligent work. When the dream, which was entitled "All Sorts and Conditions of Men," had run for twelve months in the magazine, it was published as "An Impossible Story" and, lo! in three years the impossibility had become a recognised and material fact—the "Palace of Delight" Angela Messenger planned; the People's Palace that we know. The Queen went down to Mile End to open it, and the Prince and Princess of Wales have taken a lively interest in its welfare. It is worthy of note, too, that in spite of the excitement and fatigue of the Jubilee festivities, their Royal Highnesses found time to visit the London Hospital, the great institution to which those who may frequent the People's Palace when they are well, are taken when they are ill.

The Great Northern Central Hospital.

Since the period of the Jubilee the ravages of death, with the sad ceremonies attendant on these, have once and again interfered with public acts contemplated by their Royal Highnesses, but their visit to North London last summer to open the Great Northern Central Hospital—another new experiment in hospital architecture—shows that the interest they have so long shown in the welfare of their future subjects still exists and is likely to continue.

We have only touched on those actions in the career of the Prince and Princess of Wales which belong in some degree to the province of The Hospital; but Mr. Burdett's volume, which is illustrated by admirable portraits of their Royal Highnesses and views of their home at Sandringham, contains much interesting information of other kinds. It deals at some length with the Prince as a landlord, in which capacity he has done much to promote agriculture in all its branches. It also shows the relation of the Prince and Princess to each other, for the Royal houses of Britain and Denmark have intermarried before now, and by several different lines their Royal Highnesses bear a distant kinship to each other. There is also a list, and full heraldic description, of the various orders possessed by the Prince, which will doubtless be interesting to the curious, and an appendix giving a complete list of their Royal Highnesses' public appearances since their marriage. As a book of reference it is therefore of value, and may save some future historian the labour of searching through many a file of old newspapers. But besides this, it is distinctly readable.

